# Local uterine Ang-(1–7) infusion augments the expression of cannabinoid receptors and differentially alters endocannabinoid metabolizing enzymes in the decidualized uterus of pseudopregnant rats

**DOI:** 10.1186/1477-7827-13-5

**Published:** 2015-01-17

**Authors:** K Bridget Brosnihan, Victor M Pulgar, Patricia E Gallagher, Liomar AA Neves, Liliya M Yamaleyeva

**Affiliations:** Hypertension and Vascular Research Center, Wake Forest School of Medicine, Winston-Salem, NC USA; Physiology and Pharmacology, Wake Forest School of Medicine, Winston-Salem, NC USA; Department of Obstetrics & Gynecology, Wake Forest School of Medicine, Winston-Salem, NC USA; Biomedical Research Infrastructure Center, Winston Salem State University, Winston-Salem, NC USA

**Keywords:** Pregnancy, Implantation, Decidualization, Anandamide, 2-arachidonoylglycerol, Angiotensin-(1–7), Pseudopregnancy

## Abstract

**Background:**

Endocannabinoids (ECs) are important contributors to implantation and decidualization and are suppressed in early pregnancy. Elevated levels of anandamide (AEA), the endogenous ligand for the CB1 and CB2 receptors (R), interfere with receptivity of the blastocyst. Ang-(1–7) is down-regulated in the implantation site (IS) in normal pregnancy at day 7 of gestation. We determined the effects of intra-uterine angiotensin-(1–7) [Ang-(1–7)] (24 microg/kg/h) or vehicle given into the left uterine horn on the ECs in the decidualized uterus.

**Methods:**

Ovariectomized rats were sensitized for the decidual cell reaction by steroid treatment and decidualization was induced by a bolus of oil injected into the left horn; the right horn served as a control.

**Results:**

Decidualization increased endometrial permeability (3.1+/−0.2 vs. 7.1+/−0.5 uterus/muscle of cpm of (125)I-BSA, p < 0.0001). VEGF mRNA was increased by the decidualization (1.4-fold, p < 0.05) and by Ang-(1–7) (2.0-fold, p < 0.001). CB1R mRNA was reduced by decidualization (2.7-fold, p < 0.001), but increased by Ang-(1–7) (1.9-fold, p < 0.05)**.** CB2R mRNA was increased by decidualization (4-fold, p < 0.05) and by Ang-(1–7) (2.4-fold, p < 0.001). The enzyme metabolizing AEA, fatty acid amide hydrolase (FAAH), was reduced by decidualization (7.8 fold, p < 0.001) and unchanged by Ang-(1–7) (p > 0.05), whereas the enzyme metabolizing 2-arachidonoylglycerol, monoacyl glycerol lipase (MAGL), was unchanged by decidualization (p > 0.05) and increased by Ang-(1–7) (1.7 fold, p < 0.001).

**Conclusions:**

These findings report for the first time that Ang-(1–7) augments the expression of CB1R, CB2R and MAGL in the decidualized uterus and thus may interfere with the early events of decidualization.

## Background

The outcome of pregnancy depends on the success of implantation and placentation. In previous studies, we discovered that in early normotensive pregnancy the renin-angiotensin system (RAS) [Ang–(1–7) and Ang II] was down-regulated in the uterus as compared to virgin animals and in the implantation site (IS) as compared to the adjacent interimplantation site (IIS) at day 7 of gestation [1]. The observation of reduced Ang-(1–7) and Ang II in the decidualized horn of the pseudopregnant rat further confirmed that a down-regulation of RAS is important for the early stages of decidualization [2]. In human placenta at the first trimester of aborted pregnancy, we demonstrated that Ang-(1–7) was increased [3], suggesting that an increase in Ang-(1–7) in the early uteroplacental unit is detrimental. Similar to the down-regulation of Ang II and Ang-(1–7) at early pregnancy, low levels of anandamide (AEA), an endogenous endocannabinoid, are associated with the IS as compared to the IIS and are required for the receptivity of the blastocyst’s attachment to the uterus [4]. High levels of AEA cause embryotoxicity, reduced trophoblast proliferation, and implantation failure [4–7]. The striking similarity of the pattern of distribution of the two systems (endocannabinoid system (ECS) and RAS) in early events of pregnancy and their required down-regulation during normal pregnancy makes a compelling argument to compare their regulation in the early events of pregnancy when the balance between the two systems is disrupted.

Our hypothesis is that Ang-(1–7) exerts an important regulatory role on the endocannabinoid system in the decidualization process of early gestation. In order to uncover this role, Ang-(1–7) was infused locally into one decidualized uterine horn of a pseudopregnant rat and its effects on the expression of the endocannabinoid receptors, CB1R and CB2R, and the enzyme metabolizing AEA, fatty acid amide hydrolase (FAAH), and the enzyme metabolizing another endogenous endocannabinoid, 2-arachidonoylglycerol (2-AG), monoacyl glycerol lipase (MAGL), were evaluated, together with other markers of decidualization.

## Methods

### Surgical procedures

**Figure 1:**
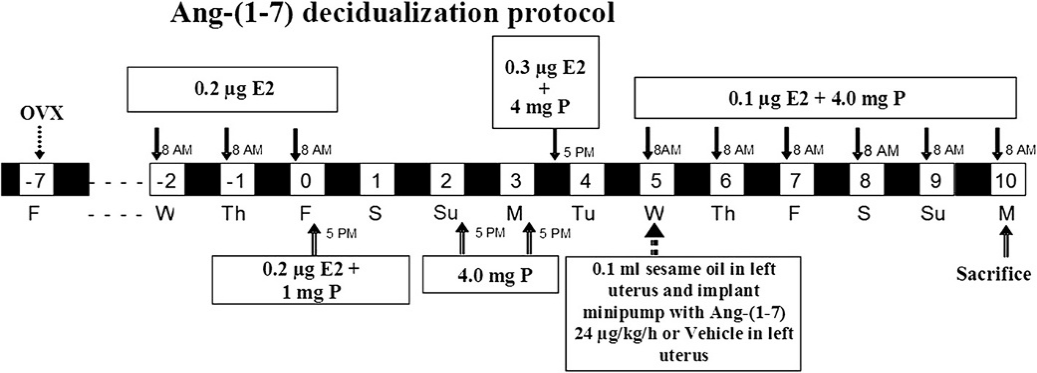
**Time course of the sequence of hormone administration to ovariectomized rats and the timing of Ang-(1–7) or vehicle administration.** Black areas represent periods of night time; numbers within light box areas indicate days relative to ovariectomy when hormones were injected subcutaneously. Arrows projecting from dose and type of hormone in boxes indicate the equivalent type of hormone that was administered sequentially. The time of day of injection is indicated. Decidualization induction and implantation of the miniosmotic pump occurred on day 5, and animals were sacrificed on day 10. E2 = 17-beta estradiol, P = progesterone.

All procedures were approved by the Wake Forest School of Medicine Animal Care and Use Committee. Female Sprague–Dawley rats (n = 9-10/group) were obtained from Harlan Laboratories at 10 weeks of age and were ovariectomized under 2% isofluorane anesthesia. Five days after surgery animals were treated with a hormone regime [17-beta estradiol (0.1. 0.2, or 0.3 μg) and progesterone (1 or 4 mg)] as illustrated in Figure 1 and as described [8]. On day 5 as indicated on Figure 1, animals were anesthetized with 2% isoflurane and 0.1 ml of sesame oil was injected into the left uterine horn; an osmotic minipump (model 2ML2, pumping rate of 5 μL/hr) was placed in the left uterine horn for delivery of either 24 μg/kg/h of Ang-(1–7) in sterile phosphate sodium buffer (PBS, pH 7.4) or vehicle. PE60 tubing attached to the minipump was inserted into the uterus lumen until it reached a plastic cuff placed 1 mm from the tip of the catheter. Sutures secured the catheter (before and after the cuff) and either Ang-(1–7) or PBS was infused. The right horn was not injected or infused and served as a control. After five days of treatment, animals were euthanized by decapitation and trunk blood was collected in a cocktail of inhibitors as previously described [9]. The non-infused and infused uterine horns were removed, weighed, snap-frozen on dry ice for mRNA analysis, or fixed in 10% neutral buffered formalin solution for immunostaining.

### Plasma angiotensins

Angiotensin (Ang) I, Ang II, and Ang-(1–7) peptides were measured in plasma by three radioimmunoassays as previously characterized [10, 11].

### Uterine permeability

A separate group of animals similarly treated was prepared for permeability studies. On the morning of day 10 the animal’s weight was recorded. Animals were anesthetized with 2% isoflurane, and 8×10(6) cpm/250 g of (125)I-BSA was administered by intracardiac injection of 0.2 mL of (125)I-BSA. The animals were allowed to recover for 15 minutes and then euthanized by decapitation. The infused and non-infused uterine horn and the ventral gastrocnemius skeletal muscle were dissected free of any fat and weighed and the amount of radioactivity was determined. The uterine permeability index was calculated as the ratio of uterine tissue radioactivity (cpm/g) to muscle tissue radioactivity (cpm/g).

### RNA isolation and Real-Time RT-PCR

Total RNA, isolated using the Trizol reagent (GIBCO, Carlsbad, CA), was incubated with AMV reverse transcriptase as previously described [12]. The primer/probe sets for CB1R, CB2R, FAAH, MAGL, vascular endothelial growth factor A (VEGF-A), apoptotic protease activating factor 1 **(**APAF), caspase 3, and caspase 9 were purchased from Applied Biosystems (Grand Island, NY). All reactions were performed in triplicate and 18S ribosomal RNA, amplified using the Taqman Ribosomal RNA control kit, served as an internal control. The results were quantified as Ct values, where Ct is defined as the threshold cycle of PCR at which the amplified product is first detected.

### Immunohistochemistry

After fixation in formalin and ethanol, uterine sections were embedded in paraffin and cut into 5-μm sections. Immunostaining was performed using the Avidin Biotin Complex (ABC) method with 0.1% diaminobenzene solution used as the chromogen as described previously [13]. Staining required antigen retrieval treatment with sodium citrate buffer (pH 6.0) at 90-95°C for 30 min. Non-specific binding was blocked in a buffer containing 10% normal goat serum, 1% Triton-X in PBS for 30 min. The uteri were incubated with the rabbit monoclonal anti-vimentin primary antibody (dilution: 1:500; Abcam, Cambridge, MA, USA) and secondary biotinylated goat anti-rabbit antibody (dilution: 1:400; Vector Laboratories, Burlingame, CA, USA). Vimentin staining was analyzed using Adobe Photoshop 7.0. Data were normalized to the intensity of the background and to the maximal value of the RGB component and reported as relative intensity units.

### Statistics and data analysis

Data were analyzed with a standard two-way analysis of variance (ANOVA) followed by the Bonferroni’s post hoc test. The Student’s t test for unpaired observations was used when appropriate (GraphPad Software, San Diego, CA). A p value of less than 0.05 was considered statistically different. All data are presented as mean +/− SEM.

## Results

**Figure 2:**
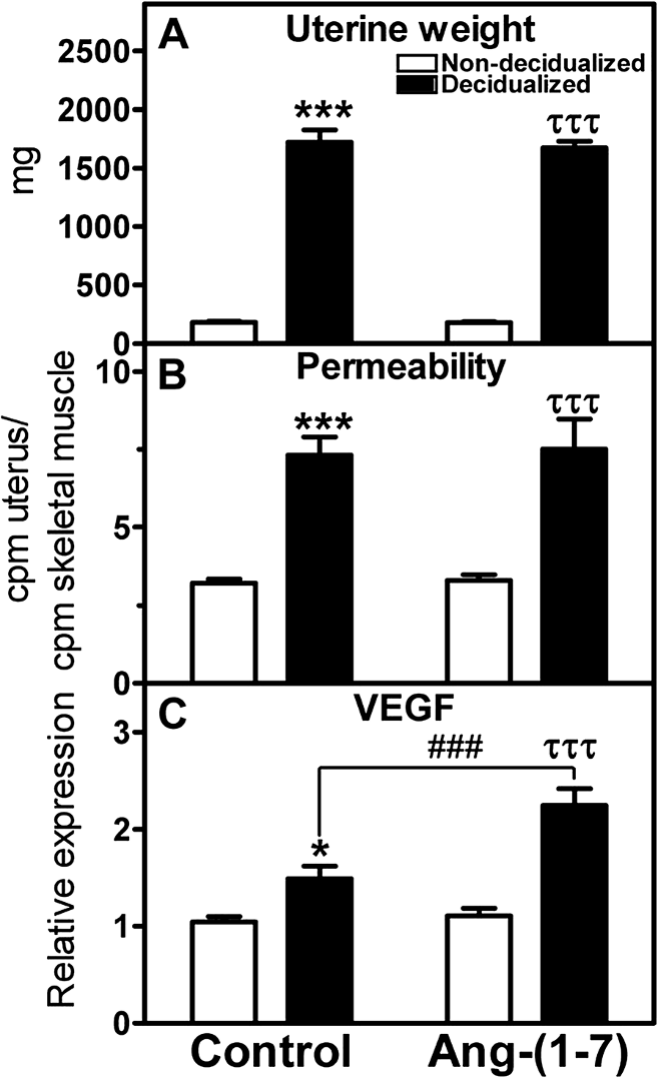
**Effects of local intra-uterine infusion of Ang-(1–7) in the decidualized horn on uterine weight (A), permeability (B), and VEGF relative gene expression (C) in non-decidualized vs decidualized uterus.** Data are expressed as mean +/− SEM. n = 9–10. ^*^p < 0.05, ^***^p < 0.001 vs. non-decidualized control; ^###^p < 0.001 vs. decidualized control; ^τττ^p < 0.001 vs. non-decidualized in the Ang-(1–7) treated animal.

**Figure 3:**
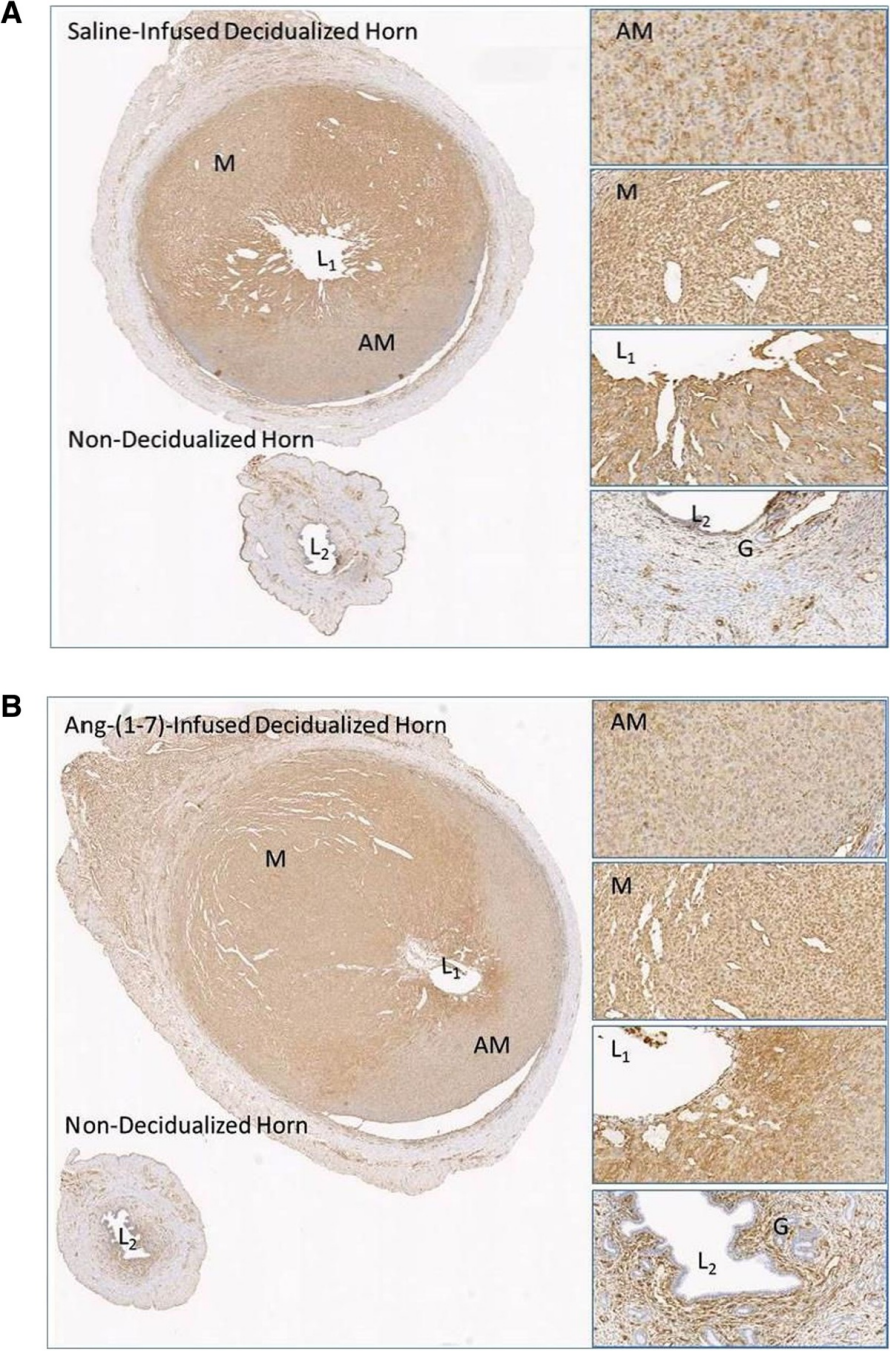
**The effect of local Ang-(1–7) infusion in the decidualized horn on vimentin staining.** Panel **A** shows vimentin staining of the saline-infused decidualized horn. Panel **B** shows vimentin staining of the Ang-(1–7)-infused decidualized horn. Magnification: 2x for the uterine horn images, 20x for the images of mesometrial (M) pole, anti-mesomentrial (AM) pole, uterine lumen of infused horn (L1) and uterine lumen non-infused horn (L2). G, endometrial glands.

**Figure 4:**
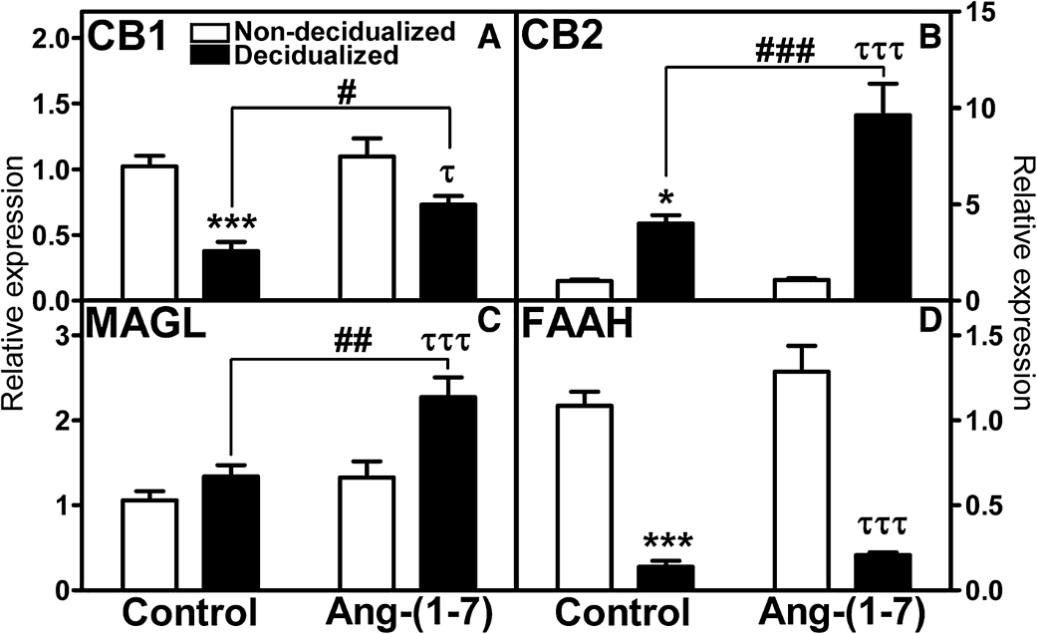
**Effects of local intra-uterine infusion of Ang-(1–7) in the decidualized horn on relative gene expression of CB1R (A), CB2R (B), MAGL (C), and FAAH (D) in non-decidualized vs decidualized uterus.** Data are expressed as mean +/− SEM. n = 9–10. ^*^p < 0.05, ^***^p < 0.001 vs. non-decidualized control; ^#^p < 0.01, ^##^p < 0.01, ^###^p < 0.001 vs. decidualized control; ^τ^p < 0.05, ^τττ^p < 0.001 vs. non-decidualized Ang-(1–7) treated animal.

**Figure 5:**
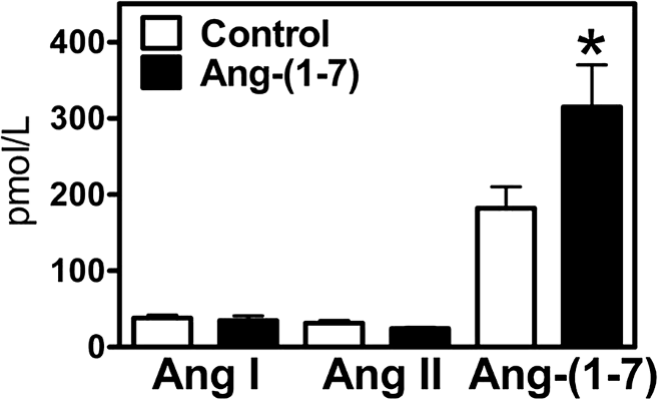
**Effects of local intra-uterine infusion of Ang-(1–7) in the decidualized uterus on plasma levels of angiotensin peptides.** Plasma levels of Ang I, Ang II, Ang-(1–7) are shown in control and Ang-(1–7)-treated animals. Data are expressed as mean +/− SEM. n = 9–10. ^*^p < 0.05, vs. control.

Figure 2 compares the uterine weight and permeability levels in the decidualized uterine horn in the presence or absence of Ang-(1–7) local infusion. There was a 9.6 fold increase in uterine weight in the oil-induced decidualized horn as compared to the non-decidualized horn, which was not altered by Ang-(1–7) infusion. One of the first events of decidualization is an increase in permeability. Permeability measured as the ratio of radiolabeled I(125)-albumin in the uterus relative to the radioactivity of skeletal muscle albumin increased with decidualization, but this increase was unchanged with Ang-(1–7) treatment. VEGF, also known as vascular permeability factor, was increased with decidualization; in the presence of Ang-(1–7) treatment VEGF mRNA expression was augmented 2-fold in the decidualized horn as compared to the control decidualized horn. There was no effect of decidualization or Ang-(1–7) treatment on markers of apoptosis, including caspase 3, caspase 9, and APAF mRNA (Table 1). In addition, uterine tissue was immunostained for vimentin (Figure 3A and B). There was a clear difference in intensity of vimentin staining between the anitmesometrial and mesometrial poles (Figure 3A and B). There was no difference in the antimesometrial staining between treated and non-treated uteri. Vimentin staining was evident in the lining of the vascular components of the mesometrium of the decidulized uterus and was similar in Ang-(1–7)-infused vs. PBS-infused decidualized uterine horns (PBS-infused horn: 0.33+/−0.04 vs. Ang-(1–7)-infused horn: 0.28+/−0.04 relative intensity of vimentin staining, n = 3–5, p > 0.05).Decidualization is characterized by a 2.7–fold decrease in CB1R mRNA levels, 4-fold increase in CB2R mRNA, 7.8 fold decrease in FAAH mRNA, and no significant change in MAGL mRNA levels (Figure 4A-D). The Ang-(1–7) treated decidualized horn (Figure 4A) resulted in a 1.9-fold increase in CB1R mRNA levels as compared to the control decidualized horn; the Ang-(1–7) treated horn, however, remained significantly lower than the non-decidualized horn. The Ang-(1–7) treatment resulted in an augmented 2.4-fold increase in CB2R mRNA levels over the control decidualized horn (Figure 4B); the Ang-(1–7)-treated decidualized horn remained 9-fold higher than the non-decidualized horn. Ang-(1–7) treatment increased the expression of MAGL mRNA by 1.7-fold over its non-decidualized control and by 1.7-fold over the decidualized control (Figure 4C). Although there was a significant reduction in FAAH mRNA levels with decidualization, this reduction was unchanged with Ang-(1–7) treatment in the decidualized horn (Figure 4D).Circulating levels of Ang I, Ang II, and Ang-(1–7) were measured in control and Ang-(1–7) treated pseudopregnant rats. Circulating levels of Ang I and Ang II were unchanged by the intra-uterine infusion of Ang-(1–7) (Figure 5), but there was an increase in circulating levels of Ang-(1–7) with the local uterine Ang-(1–7) infusion.

**Table 1:** **Effects of local intra-uterine infusion of Ang-(1–7) into a decidualized horn on the relative gene expression of markers of apoptosis, APAF, caspase 3, and caspase 9 in non-decidualized vs. decidualized uterus**

	Control	Control	Ang-(1–7)	Ang-(1–7)
	Non-decidualized	Decidualized	Non-decidualized	Decidualized
**APAF**	1.05 +/− 0.08	1.07 +/− 0.12	1.00 +/− 0.08	1.28 +/− 0.13
**Caspase 3**	1.01 +/− 0.11	1.17 +/− 0.13	1.20 +/− 0.10	1.13 +/− 0.14
**Caspase 9**	1.05 +/− 0.08	1.25 +/− 0.10	1.08 +/− 0.94	1.29 +/− 0.13

Values and mean +/− SEM, n = 9–10. APAF **=** apoptotic protease activating factor 1.

## Discussion

Decidualization is an important step whereby the maternal uterus is remodeled. In addition, the decidual cells are important players in the recognition of implanting a viable embryo. Although decidualization usually occurs in conjunction with receiving the implanting blastocyst, mechanical stimulation to the uterine luminal surface in pseudopregnant rodents can induce differentiation of uterine stromal cells into decidual cells, in a manner that is similar to blastocyst implantation. Endocannabinoids [AEA and 2-AG] are important components of this process with low levels of AEA and 2-AG being associated with the implantation site (IS) and being required for the receptivity of the blastocyst’s attachment to the uterus [4]. However, high levels of AEA cause embryotoxicity, reduced trophoblast proliferation, and implantation failure [5, 14, 15]. Gene targeting experiments demonstrated that CB1R is essential for implantation [4], decidualization, and embryo development [7]. The role for CB2R is not as clearly established in early pregnancy. In previous studies, we discovered that in early normotensive pregnancy the RAS [Ang–(1–7) and Ang II] was down-regulated in the uterus compared to virgin rats, in the implantation site compared to the adjacent interimplantation site [1], and in the decidualized horn of the pseudopregnant rat [2]. Thus, in this study we asked the question of whether locally infused Ang-(1–7) in the decidualized horn would alter the endocannabinoid system. Our data demonstrated that Ang-(1–7) infusion resulted in an up-regulation of CB1R, CB2R, and MAGL mRNA in the decidualized uterine horn as compared to the non-treated decidualized horn. The down-regulated FAAH mRNA with decidualization was unchanged by Ang-(1–7) treatment.

In the pseudopregnant rat, CB1R is significantly decreased in the decidualized uterus. Sun et al. [7] have demonstrated that lower levels of CB1R are beneficial for implantation and decidualization. The reduction in CB1R mRNA is consistent with the lower levels of AEA, which has been reported in the decidualized uterus [4, 16]. In the presences of Ang-(1–7) we showed up-regulation of CB1R expression in the decidualized uterus. The studies of Moghadam al [14] demonstrated that activation of the CB1R inhibited decidualization using an *in vitro* system of induction of decidualization, suggesting that higher levels of AEA and its level of binding to the CB1R would be harmful to decidualization and implantation.

Our studies are the first to demonstrate up-regulation of CB2R mRNA in the decidualized uterus of the pseudopregnat rat. *In vitro* studies demonstrate that embryos from CB2R knockout mice treated with AEA show similar aberrant development as wild type, indicating CB2R is not required for early development [17]. The average litter size of CB2R knockout mice was normal [7]. A number of studies using pharmacological blockade have shown the importance of CB1R but not CB2R in early pregnancy. Studies assessing the rate of zona hatching of blastocyst *in vitro* provided evidence that the AEA effects were abolished by a CB1R antagonist, SR141716A, but not the CB2R antagonist, SR144528 [18]. Similarly CB1R inactivation induces pre-term labor, whereas CB2R inactivation does not [19]. The exact role of CB2R at early pregnancy is yet to be defined, but the overall assessment is that the majority of early pregnancy events are mediated by CB1R rather than CB2R. Our finding of an increase in CB2 receptor with Ang-(1–7) treatment in the decidualized horn most likely would contribute to the imbalance between Ang-(1–7) and the endocanabinoid system.

The reduction in FAAH mRNA in the decidualized uterus is consistent with reports of reduced FAAH activity in the implantation site of early pregnancy [5, 20] and in the pseudopregnant uterus [4, 21]. Based on the FAAH knockout animals, the levels of AEA, but not 2-AG, were increased in the placenta [5, 7], indicating that FAAH is the major degrading enzyme for AEA. Thus, the reported reduced levels of AEA in the decidualized uterus and implantation site may be more dependent on reduced anadamide synthase or NAPE-PLD, which parallels the reduction in AEA in the implantation site and the pseudopregnant uterus [21]. Our studies showed no effect of Ang-(1–7) on the expression of FAAH in the decidualized uterus, and these findings would be consistent with a lack of change on FAAH-dependent AEA levels with Ang-(1–7) treatment. Furthermore, the concentration difference of AEA (pmol/g) and 2-AG (nmol/g) in the implantation and interimplantation sites of early pregnancy and pseudopregnancy would suggest that 2-AG may be the dominate endocannabinoid that varies with Ang-(1–7) treatment.

MAGL mRNA was not changed with decidualization. These findings differ from Wang et al. [5] who showed that MAGL immunocytochemical expression was induced in the implantation site with lower levels of expression in the interimplantation site. Their finding is consistent with MAGL in the implantation site participating in a protective role of the embryo from excessive levels of 2-AG. Because MAGL is the main enzyme responsible for the degradation of 2-AG and because it has been suggested that 2-AG levels are important for progression of pregnancy [22, 23], our studies showing unchanged levels of MAGL with decidualization suggests that 2-AG in the pseudopregnant decidualized uterus would not be changing in an MAGL-dependent manner. On the other hand, MAGL expression increased with Ang-(1–7) treatment in the decidualized uterus. This finding is consistent with increased degradation of 2-AG with Ang-(1–7) treatment in the decidualized horn and thus would have an effect on the balance of 2-AG/AEA by reducing the levels of 2-AG. However, this suggestion depends on the measurement of MAGL protein and activity levels in association with the levels of 2-AG between the decidualized and non-decidualized horns.

In these studies we used a number of markers to assess the state of the pseudopregnant rat, including permeability, uterine weight, VEGF, apoptotic markers and immunostaining of vimentin. In the decidualized horn, we demonstrated increases in uterine weight and permeability that were associated with an increase in VEGF and a decrease in CB1R. Ang-(1–7) treatment did not alter uterine weight and permeability, even though the expression of CB1R was increased. One would have expected that uterine weight and permeability would be influenced by an increased expression of CB1R. It appears that these biochemical changes were not strong enough to affect the permeability and uterine weight changes that occurred with decidualization. One possibility that needs to be considered is that if these biochemical changes occurred in the implantation site they may elicit a local change in permeability relative to the immediately adjacent interimplantation site. This is clearly a limitation of the study. In addition, because we saw changes in VEGF and not permeability, this may suggest that the changes of permeability have peaked and cannot increase further. Although VEGF is known to be a potent angiogenic factor, it was first characterized as a permeability factor [24]. Its increase with decidualization has been previously described [25]. With *in situ* hybridization of VEGF mRNA, Halder et al. [25] described its distribution in the luminal epithelium and decidualizing stroma surrounding the blastocyst and with progression of pregnancy its accumulation in decidual cells in both mesometrial and antimesometrial poles of the uterus. VEGF and its receptors expression were more intense in the mesometrial pole, which is the presumptive site of placentation and heightened angiogenesis. In our study VEGF mRNA was further increased by Ang-(1–7). This is the first study to demonstrate an effect of Ang-(1–7) on VEGF during pseudopregnancy. In previous studies Ang-(1–7) has been demonstrated to inhibit angiogenesis, cause a reduction in VEGF, and inhibit tumor growth in athymic mice with human LNCaP prostate cancer cells [26] and in human lung tumor xenografts [27]. Our findings demonstrate that the process of decidualization is quite different from tumor growth in relation to VEGF’s regulation by Ang-(1–7). Although Ang-(1–7) is anti-angiogenic and tumor growth inhibitory in cancer studies, in the early stages of reproduction it promotes the increase in VEGF mRNA, suggesting a positive influence on angiogenesis under these conditions. Another early event of pregnancy is apoptosis. Our data demonstrate that markers for apoptosis including caspase 3, caspase 9, and APAF, were unchanged with decidualization and with Ang-(1–7) treatment. Our study assessed vimentin, a marker of decidualized cells. Although we were able to demonstrate clear differences in immunostaining between the mesometrial and antimesometrial regions of the decidualized horns, there was no effect of Ang-(1–7) on the intensity of staining of vimentin in the decidualized uterus. Overall, we were able to demonstrate that a number of biochemical and cellular markers were stable during Ang-(1–7) treatment, while others, including VEGF, CB1R, CB2R and MAGL, were specifically changed, indicating that they are part of the temporal spatial changes which occur at different times or are localized to specific regions during the early pregnancy events.

Ang I and Ang II were not influenced by the Ang-(1–7) infusion, however, an increase in circulating levels of Ang-(1–7) was associated with the local infusion of Ang-(1–7) in the uterus. Comparison of the biochemical changes in non-decidualized horns in animals with and without Ang-(1–7) treatment showed that there were comparable levels of gene expression, uterine weight, and permeability changes, demonstrating that this spill over into the circulation from the infused horn had no effect on the control horn. Furthermore, the levels of Ang-(1–7) in the control group were comparable to previous levels reported in pseudopregnant animals [2].

## Conclusions

The present study demonstrates that local administration of Ang-(1–7) into the decidualized uterus up-regulates a number of components of the endocannabinoid system. Because the levels of Ang-(1–7) and ECS are normally reduced during decidualization, our study suggests that alteration of one component may interfere with the balanced interaction of these two systems in the early events of pregnancy. Follow-up studies are warranted to assess the protein and lipid components to accompany the mRNA expression.

## Abbreviations

AEA: Anandamide
ang-(1–7): Angiotensin-(1–7)
Ang I: Angiotensin I
Ang II: Angiotensin II
APAF: Apoptotic protease activating factor 1
BSA: Bovine serum albumin
ECs: Endocannabinoids
ECS: Endocannabinoid system
CB1R and CB2R: Endocannabinoid receptors
FAAH: Fatty acid amide hydrolase
IS: Implantation site
IIS: Interimplantation site
MAGL: Monoacyl glycerol lipase
PBS: Phosphate sodium buffer
RAS: Renin-angiotensin system
VEGF-A: Vascular endothelial growth factor A
2-AG: 2-arachidonoylglycerol.
